# Live vaccines—a short‐cut to cancer viro‐immunotherapy

**DOI:** 10.15252/emmm.201911496

**Published:** 2019-11-20

**Authors:** Thomas C Wirth, Julia Niemann, Florian Kühnel

**Affiliations:** ^1^ Deparment of Gastroenterology, Hepatology, and Endocrinology Hannover Medical School Hannover Germany

**Keywords:** Cancer, Immunology

## Abstract

Tumour immunotherapies have been a breakthrough in clinical oncology but only a few patients benefit from this progress. Additional interventions that sensitize immunologically cold tumours for the administration of checkpoint modifiers are urgently needed. In this issue of *EMBO Molecular Medicine*, Aznar *et al* present the already approved yellow fever vaccine 17D as an oncolytic agent for tumour immunoactivation. In tumour‐bearing mice, they demonstrated a convincing synergy of the vaccine with CD137 agonistic antibodies resulting in significantly improved survival.

## Graphical abstract

In this Issue of *EMBO Molecular Medicine*, Aznar *et al* demonstrate the therapeutic potential of the already approved yellow fever vaccine 17D for viro‐immunotherapy of cancer.

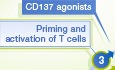

CTLA‐4 and PD‐1/PD‐L1 checkpoint immunotherapy has significantly improved long‐term survival in metastatic melanoma, and clinical responses have been observed across a broad range of tumour entities. The experience that novel agents are indeed capable of facilitating durable tumour responses in advanced cancers has been paradigm‐shifting and fuelled new phantasy for the future of cancer therapy. However, from a more rational perspective, these clinical studies unequivocally demonstrated that only a small subset of patients responds in this spectacular manner, whereas the vast majority does not benefit.

Some characteristics have been linked to tumour sensitivity to checkpoint immunotherapy. For example, PD‐L1 expression in tumours indicating immune activity, or a high mutational burden as a source of neoantigens, increases the probability that a tumour will respond to therapy. However, regarding the enormous genetic diversity of tumours and the complex array of mechanisms a tumour can employ to keep the host's immune defence in check, it is much more difficult to understand why tumours do not respond.

Critical steps to establish antitumour immunity include the release of tumour antigen, antigen presentation and T‐cell priming. Furthermore, migration to the tumour and infiltration of tumour tissue are required that T cells recognize and kill tumour cells again to restart the cancer immunity cycle. Tumours can be classified according to essential stages where this cycle might be dysfunctional (Chen & Mellman, 2017). While “inflamed” (or “hot”) tumours show signs of immune activity and have highest probability to respond to checkpoint inhibition, immune‐inactive (“cold”) tumours are either “immune‐excluded” due to defects in trafficking and tumour infiltration or “immune‐desert” with dysfunctional antigen presentation and T‐cell priming. The PD‐1/PD‐L1 axis is therefore not unique in restraining the antitumour response. In these cold tumours, other critical dysfunctions need to be addressed to keep the cancer immunity cycle going. Consequently, there is an intense search for interventions or agents that provide essential help to convert cold tumours into hot ones and enable effective checkpoint immunotherapy.

Oncolytic viruses that preferentially replicate in and lyse tumour cells are promising tools in this regard. A mutant herpesvirus, T‐Vec, has been approved in 2015 following positive clinical trial data (Andtbacka *et al*, 2015). In general, viral oncolysis leads to effective release of tumour antigen and T‐cell priming. Virus‐induced tumour inflammation allows for T‐cell infiltration and further killing of cancer cells. Since these functions positively affect the cancer immunity cycle at critical stages, oncolytic viruses are assumed to be ideally suited to initiate and to propel the cycle. In experimental models, oncolytic viruses synergize with systemic CTLA‐4 or PD‐1 blockade to achieve effective antitumoural immunity (Zamarin *et al*, 2014; Woller *et al*, 2015). Clinical studies using T‐Vec and the PD‐1 inhibitor pembrolizumab are ongoing (Ribas *et al*, 2017).

However, regarding all needed basic research investigations, preclinical development, toxicology studies and regulatory affairs, it is a tedious process to carry an oncolytic virus from the hypothesis to clinic trials, which can easily consume half of the professional lifetime of a researcher. Therefore, a smart idea is to exploit already approved live virus vaccines, which can exert critical functions of a bona fide oncolytic virus (Fig 1).

**Figure 1:**
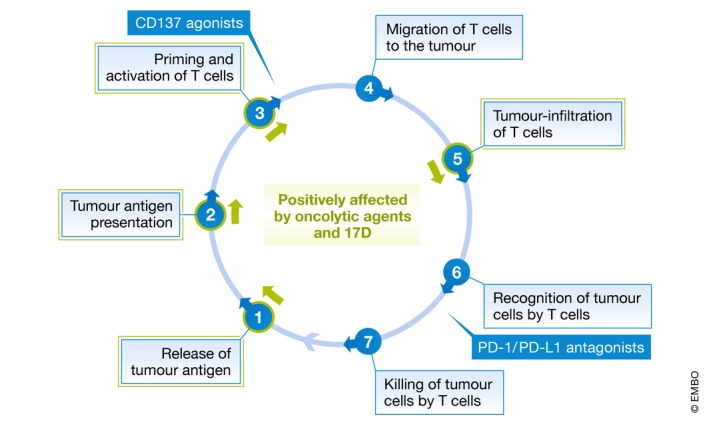
The yellow fever vaccine 17D facilitates tumour immunotherapy when applied as an oncolytic agent Stages of the cancer immunity cycle that are supported by oncolytic agents are marked by green arrows.

In the current issue of *EMBO Molecular Medicine*, Aznar *et al* (2019) have repurposed the established yellow fever vaccine 17D for tumour immunotherapy. The live, attenuated virus strain 17D is used as a prophylactic vaccine that provides potent protection against the wild‐type virus. Aznar *et al* demonstrated that 17D replicates in and kills a broad range of human and mouse tumour cells thus qualifying the vaccine as an oncolytic agent. Furthermore, they showed in syngeneic murine models bearing subcutaneous tumours that intratumoural application of the vaccine did not only delay the growth of treated tumours but also yielded an abscopal effect on contralateral, untreated tumours in a CD8 T‐cell‐dependent manner. This observation demonstrated that the vaccine induced antitumour CD8 T cells, an essential step for long‐term responses and for the success of immune checkpoint modifiers. Accordingly, the authors investigated a possible synergy triggered by these interventions. Whereas a rather modest enhancement of PD‐1 checkpoint therapy was observed, the synergy with an agonistic CD137 antibody was striking and led to dramatically improved survival. These results confirm that 17D can be successfully used to prepare a tumour for administration of immune checkpoint modifiers. The findings are consistent with previous results showing that a genetically engineered oncolytic vaccinia virus in combination with a CD137 antibody was more potent compared to monotherapies (John *et al*, 2012). Promising alternative approaches to optimize intratumoural T‐cell activation have used oncolytic viruses to directly express checkpoint inhibitors or BiTEs (bispecific T‐cell engagers) (Engeland *et al*, 2014; Freedman *et al*, 2017).

Preexisting antibodies against yellow fever may be a limitation for the 17D vaccine. However, these should be rather low in most developed countries. Moreover, the assumption that preexisting antibodies are a limitation factor for oncolytic viruses is currently challenged by both practical considerations and opposing observations. Indeed, the influence of neutralizing antibodies is a minor factor when the virus is directly injected into the tumour. Moreover, Aznar *et al* found a higher antitumoural efficacy of 17D in mice that had been preimmunized, which confirms previous results by Ricca *et al* (2018) who observed that intratumoural treatment with Newcastle disease virus was more effective in mice with preexisting immunity. The underlying mechanisms of these counterintuitive findings remain enigmatic and deserve further investigations.

In summary, Aznar *et al* showed that the 17D vaccine is a promising “ready‐to‐use” oncolytic agent that would strengthen the effect of checkpoint modifiers and may significantly accelerate the establishment of viro‐immunotherapy.

## References

[emmm201911496-bib-0001] Andtbacka RH , Kaufman HL , Collichio F , Amatruda T , Senzer N , Chesney J , Delman KA , Spitler LE , Puzanov I , Agarwala SS *et al* (2015) Talimogene laherparepvec improves durable response rate in patients with advanced melanoma. J Clin Oncol 33: 2780–2788 26014293 10.1200/JCO.2014.58.3377

[emmm201911496-bib-0002] Aznar MA , Molina C , Teijera A , Rodriguez I , Azpilikueta A , Garasa S , Sanchez‐Paulete AR , Cordeiro L , Etxeberria I , Alvarez M *et al* (2019) Repurposing the yellow fever vaccine for intratumoral immunotherapy. EMBO Mol Med 12: e10375 10.15252/emmm.201910375 PMC6949490 31746149

[emmm201911496-bib-0003] Chen DS , Mellman I (2017) Elements of cancer immunity and the cancer‐immune set point. Nature 541: 321–330 28102259 10.1038/nature21349

[emmm201911496-bib-0004] Engeland CE , Grossardt C , Veinalde R , Bossow S , Lutz D , Kaufmann JK , Shevchenko I , Umansky V , Nettelbeck DM , Weichert W *et al* (2014) CTLA‐4 and PD‐L1 checkpoint blockade enhances oncolytic measles virus therapy. Mol Ther 22: 1949–1959 25156126 10.1038/mt.2014.160 PMC4429737

[emmm201911496-bib-0005] Freedman JD , Hagel J , Scott EM , Psallidas I , Gupta A , Spiers L , Miller P , Kanellakis N , Ashfield R , Fisher KD *et al* (2017) Oncolytic adenovirus expressing bispecific antibody targets T‐cell cytotoxicity in cancer biopsies. EMBO Mol Med 9: 1067–1087 28634161 10.15252/emmm.201707567 PMC5538299

[emmm201911496-bib-0006] John LB , Howland LJ , Flynn JK , West AC , Devaud C , Duong CP , Stewart TJ , Westwood JA , Guo ZS , Bartlett DL *et al* (2012) Oncolytic virus and anti‐4‐1BB combination therapy elicits strong antitumor immunity against established cancer. Cancer Res 72: 1651–1660 22315352 10.1158/0008-5472.CAN-11-2788

[emmm201911496-bib-0007] Ribas A , Dummer R , Puzanov I , VanderWalde A , Andtbacka RHI , Michielin O , Olszanski AJ , Malvehy J , Cebon J , Fernandez E *et al* (2017) Oncolytic virotherapy promotes intratumoral T cell infiltration and improves anti‐PD‐1 immunotherapy. Cell 170: 1109–1119 28886381 10.1016/j.cell.2017.08.027 PMC8034392

[emmm201911496-bib-0008] Ricca JM , Oseledchyk A , Walther T , Liu C , Mangarin L , Merghoub T , Wolchok JD , Zamarin D (2018) Pre‐existing immunity to oncolytic virus potentiates its immunotherapeutic efficacy. Mol Ther 26: 1008–1019 29478729 10.1016/j.ymthe.2018.01.019 PMC6079372

[emmm201911496-bib-0009] Woller N , Gurlevik E , Fleischmann‐Mundt B , Schumacher A , Knocke S , Kloos AM , Saborowski M , Geffers R , Manns MP , Wirth TC *et al* (2015) Viral infection of tumors overcomes resistance to PD‐1‐immunotherapy by broadening neoantigenome‐directed T‐cell responses. Mol Ther 23: 1630–1640 26112079 10.1038/mt.2015.115 PMC4817928

[emmm201911496-bib-0010] Zamarin D , Holmgaard RB , Subudhi SK , Park JS , Mansour M , Palese P , Merghoub T , Wolchok JD , Allison JP (2014) Localized oncolytic virotherapy overcomes systemic tumor resistance to immune checkpoint blockade immunotherapy. Sci Transl Med 6: 226ra32 10.1126/scitranslmed.3008095 PMC4106918 24598590

